# Real-Time Monocular Vision System for UAV Autonomous Landing in Outdoor Low-Illumination Environments

**DOI:** 10.3390/s21186226

**Published:** 2021-09-16

**Authors:** Shanggang Lin, Lianwen Jin, Ziwei Chen

**Affiliations:** 1 School of Electronic and Information Engineering, South China University of Technology, Guangzhou 510640, China; eelwjin@scut.edu.cn (L.J.); eeczw2014@mail.scut.edu.cn (Z.C.); 2 South China University of Technology-Zhuhai Institute of Modern Industrial Innovation, Zhuhai 519175, China

**Keywords:** unmanned aerial vehicle, autonomous landing, low-illumination, marker detection, real-time

## Abstract

Landing an unmanned aerial vehicle (UAV) autonomously and safely is a challenging task. Although the existing approaches have resolved the problem of precise landing by identifying a specific landing marker using the UAV’s onboard vision system, the vast majority of these works are conducted in either daytime or well-illuminated laboratory environments. In contrast, very few researchers have investigated the possibility of landing in low-illumination conditions by employing various active light sources to lighten the markers. In this paper, a novel vision system design is proposed to tackle UAV landing in outdoor extreme low-illumination environments without the need to apply an active light source to the marker. We use a model-based enhancement scheme to improve the quality and brightness of the onboard captured images, then present a hierarchical-based method consisting of a decision tree with an associated light-weight convolutional neural network (CNN) for coarse-to-fine landing marker localization, where the key information of the marker is extracted and reserved for post-processing, such as pose estimation and landing control. Extensive evaluations have been conducted to demonstrate the robustness, accuracy, and real-time performance of the proposed vision system. Field experiments across a variety of outdoor nighttime scenarios with an average luminance of 5 lx at the marker locations have proven the feasibility and practicability of the system.

## 1. Introduction

Unmanned aerial vehicles are cost-efficient, highly maneuverable, and casualties free aerial units that have been broadly adopted in civil applications and military operations, such as surveillance, traffic and weather monitoring, cargo delivery, agricultural production, damage inspection, radiation mapping, and search and rescue (SAR), to name a few [1,2,3,4]. For those missions requiring repeated flight operations where human intervention is impossible, autonomous takeoff and landing are essential and crucial capabilities for a UAV, which has been extensively studied by researchers from all over the world during the last few decades. Although launching a UAV is relatively easy, landing it is the most challenging part in many circumstances due to high risks and environmental uncertainties. According to statistics, crashes and accidents are most likely to occur in the landing phase, jeopardizing the safety of the UAVs involved. For a successful autonomous landing, a prerequisite is to know the precise location of the landing site. With this information, a UAV could gradually minimize its distance to the landing site, descend to a proper altitude, and perform touch-down in the final descent phase. To resolve this pressing issue, a widely accepted approach is to use machine vision to detect artificial landing markers for assisting UAV autonomous landing. One of the most significant advantages of machine vision is that it provides rich information about the surrounding environments without emitting radiation. It is also lightweight, low-cost, energy consumption efficient, and friendly to stealth operations. Moreover, machine vision is robust to signal jamming and telemetry interference due to its passive nature. At a distance, a UAV may carry out preliminary detections on landing markers using machine vision, while relying on other navigational means such as the global navigation satellite system (GNSS) or an inertial measurement unit (IMU) [5]. At close range, vision sensors can determine both the relative positions and attitudes between the UAV and the landing marker within sub-millimeter accuracy [6], information of which is essential for precise landing control. During the entire landing maneuver, vision sensors can couple with GNSS or IMU to obtain more reliable measurements.

To date, there exist many studies describing vision-based methods for UAV autonomous landing in either simulations or indoor well-contained laboratory environments. They are the demonstration or proof-of-concept that could light a path toward more practical and realistic solutions [7,8]. Some of the recent studies have carried out flight tests in real-world environments where well-illuminated scenes favor the vision systems [9,10]. With the proliferation of UAV applications, there emerges an increasing need to operate UAVs at nighttime, benefiting from less airspace traffic and less human-activity-based interferences. Some countries and regions have also issued specific legal frameworks to allow for operation of a UAV at night. According to the Global Drone Regulations Database [11], for instance, the Federal Aviation Administration (FAA) in the United States brought the 2021 New FAA Drone Regulations into effect on 21 April 2021. The regulations state that a certificated operator must comply with the FAA’s training and testing requirements and apply anti-collision lighting before flying the drone at night. From 31 December 2020, the European Union Aviation Safety Agency (EASA) allowed night operations of UAVs unless the state or region defines a specific zone where night ops are not possible for security reasons. In 2016, China’s civil flight authority, the Civil Aviation Administration of China (CAAC) issued new rules to allow UAVs operating beyond visual line of sight (BVLOS) to fly outside the no-fly-zone (NFZ) in the nighttime. Recently, Australia’s Civil Aviation Safety Authority (CASA) has approved flying a drone or remotely piloted aircraft (RPA) at night for licensed operators. With the continuous improvement of laws and regulations, UAV nighttime operations will be increasingly common in the foreseeable future. Some related studies have also investigated the potential of nighttime-based autonomous navigation. However, autonomous nighttime landing is still a challenging task. Researchers have introduced supplementary means, such as applying active light sources to markers [12], or using a shipdeck-deployed infrared light array [13], so that the vision systems may see the highlighted landing spots through the darkness. Nonetheless, an obvious drawback of the active-marker-based methods is that it requires additional ground-based infrastructures to be deployed. The active nature has a strong possibility to defeat stealth operations. To our best knowledge, none of the existing approaches have reported a reliable performance of UAV autonomous nighttime landing using conventional vision sensors and non-active marker designs only. In the darkness, the onboard vision system may significantly encounter more difficulties than daytime for the following reasons: 1. Low contrast and visibility: Since very limited light is reflected on the object surfaces due to poor illumination conditions, the landing marker may have little appearance from a dark background. Target detection methods relying on contour detection, geometric analysis, and inline-texture-based feature matching may no longer be applicable owing to low contrast and visibility. 2. Partial occlusion: Shadows caused by light sources occasionally overlay on the landing marker, thus leading to partial occlusion. In addition, the marker itself may be partially or entirely out of field-of-view (FOV) due to UAV ego-motion, which is another primary reason for occlusion. 3. Motion blur and noise: Under low-illumination conditions, the exposure time should be set longer to capture a relatively brighter image. However, longer exposure time combined with camera and UAV motion results in strong motion blur, and inevitably brings noises to image acquisition. These disadvantages hinder a UAV from accomplishing autonomous landing at night.

Considering the aforementioned issues, this paper aims to close the gaps in the relative works and offer a solution towards UAV autonomous landing at nighttime without the need of an active-sourced marker, where the illumination conditions become unfavorable for a conventional vision sensor. In our previous research on UAV autonomous shipboard landing, we set a solid foundation and gained rich experience in vision-based target detection and closed-loop flight control in indoor laboratory environments [14]. In this paper, we intend to extend the vision-based approach further to accommodate the challenges of nighttime landing in outdoor environments. The significant contributions of this paper are summarized as follows:

- A novel monocular vision system is presented for landing marker localization at nighttime. It consists of a model-based scheme for low-illumination image enhancement, a hierarchical-based method consisting of a decision tree with an associated light-weight CNN for coarse-to-fine landing marker detection and validation, and a post-processing technique for keypoint extraction. Such an approach is able to perform robust and accurate landing marker detection in low-illumination nighttime scenarios from different altitude levels without lightening up the marker.
- For low-illumination image enhancement, we analyze a model-based scheme and refine the process by relaxing some of the restrictions and avoiding redundant calculation to speed up the algorithm. For landing marker detection and validation, the first three nodes of the decision tree are designed based on loose criteria, and the last lightweight CNN node maintains a strict standard of classification. Our vision system can quickly adapt to other landing marker designs by simply retraining the CNN node.
- The solution described in this paper has been verified outdoors in various field environments with an average luminance of 5 lx at the landing marker locations, feasibility, accuracy, and real-time performance of which are investigated comprehensively. To our best knowledge, none of the existing approaches have reported UAV-based landing marker detection using conventional visual sensors and non-active landing markers in such low-illumination conditions.

The remainder of this paper is organized as follows: in Section 2, the literature on relative topics is reviewed in detail; the hardware and software involved in this work are presented in Section 3; in Section 4, the algorithm for low-illumination image enhancement is elaborated; the hierarchical-based approach for landing marker detection and validation is described in Section 5; experiments and the corresponding discussion are depicted in detail in Section 6, and the paper is concluded in Section 7.

## 2. Literature Review

Previously, vision-based autonomous landing for UAVs in outdoor environments has been comprehensively studied. Most of these approaches use the onboard vision sensors to recognize a pre-defined reference object, a landing marker in general, for calculating the relative pose (position and attitude) between the UAV and the marker. The flight system then utilizes such information to perform landing maneuvers. Dated back in 2003, Saripalli, Montgomery, and Sukhatme [15] conducted the groundbreaking research for landing an autonomous unmanned helicopter outdoor on a helipad by computer vision based on calculating the image invariant moments of the landing pattern, the result of which has been broadly accepted and well-commented on by many other researchers. Since then, various marker-based proposals have been reported, in which distinctive features, such as geometrical shapes, characters, color blocks, and a combination of them have been commonly applied to design landing markers. For instance, Lee et al. [16], Serra et al. [17] and Wu et al. [18] combined black and white square-shaped tags as the landing marker to extract the relative camera position and orientation followed by image-based visual servoing (IBVS) for target tracking. In this case, the square corners are distinctive and robust enough for the visual algorithms to detect. A color-based marker design was reported in [19], where specific color-based segmentation techniques in conjunction with filtering methods and shape detection can distinguish the target from the background effortlessly. These early works have demonstrated the integration and adaptation of vision-based approaches into UAV platforms, making vision sensors the primary sensing scheme in related applications.

In real-world applications, UAVs often operate in outdoor environments and land on moving platforms such as vehicles or ground robots in multiple scenarios of interest. In contrast to static-target-based landing, pursuing a moving target and landing on it is not always a trivial task due to complex constraints, including large displacements, target out of FOV, strong motion blur, and environmental interferences. In [20], Richardson et al. incorporated an onboard visual tracker RAPiD with a Rotomotion SR20 electric-powered UAV to demonstrate integrated landing control on a flatbed truck and an unmanned ground vehicle (UGV) with slow translational motions, respectively. It used a predefined description of the object being tracked, in this case, a customized polygon, to determine the position and orientation of the object with respect to the UAV. However, the tracker cannot cope with rapid changes in illumination. Thus, the UAV can only operate in constant illumination conditions. In 2017, the Mohamed Bin Zayed International Robotics Challenge (MBZIRC) competition organized by the Khalifa University of Science in Abu Dhabi was considered a milestone in robotic society. The competition was about landing a UAV on a ground vehicle carrying a landing marker (a cross sign within a circle) moving at a maximum speed of 15 km/h. Results indicated that there still exists a large reality gap between laboratory experiments and real-world field tests. Among the very few participants who accomplished the tasks, Li et al. [21] proposed a vision-based method comprising three stages to detect the moving marker at different altitudes. Based on the geometrical features of the marker, a combination of ellipse extraction, line slope clustering, and corner detection techniques is elaborated in detail to achieve an F-measure beyond 80% with real-time performance. This study mainly focused on vision system design, but did not elucidate the integration of the entire UAV framework. Baca et al. [9] introduced their UAV system architecture design for contesting the MBZIRC competition, where a model predictive controller (MPC) along with a nonlinear feedback controller was adopted to facilitate trajectory planning and following. The vision algorithm is comprised of adaptive thresholding, fast undistortion, ellipsecross pattern detection, and relative pose estimation, details of which were comprehensively analyzed in [22]. Besides, the vision systems of other participants are revealed in [10,23,24]. Though the MBZIRC competition offered a valuable opportunity to examine the UAVs’ performance when facing challenging real-world conditions, nighttime landing was still a task beyond its consideration. With the rapid development of deep learning, convolutional neural networks have been introduced to substitute hand-crafted features for landing marker detection in [25,26], to name a few. However, in extreme low-illumination conditions, it is still debatable whether CNN can extract features effectively or not.

Runway-based landing is another scenario in which UAV autonomous landing occurs. In [27,28,29], the visual-based approaches utilize the unique perspective angle of the forward-looking cameras to detect the runway patterns, so as to determine the relative position between the aircraft and the runway for approaching and landing control, which are the most critical phases of a flight. Although the adaptation of thermal infrared (TIR) cameras is a potential solution to all-weather operation, most of these proposals still desire good visibility conditions to accomplish landing. Alternatively, some infrastructure-based systems set up on the ground provide a functional level of accuracy and availability to guide a UAV in autonomous landing. The research group of Kong et al. proposed a stereo ground-based system consisting of visible light cameras, infrared cameras, and pan-tilt-units (PTUs) to capture and track the UAV. The system feeds the relative pose information to the UAV’s onboard autopilot to generate proper landing trajectories [30,31]. A similar approach was employed by Yang et al. in [32], in which more than two near-infrared cameras are deployed to form a camera array to track and provide real-time position and velocity of the UAV. Thus, a complicated calibration procedure is essential once the system is re-deployed. Moreover, an additional infrared lamp was mounted on the nose of the UAV to facilitate target detection and tracking. Other ground-based systems use a local wireless positioning network to assure UAV localization [33,34,35]. Such an approach has the advantage of being immune to harsh weather conditions while being relatively accurate. However, the system is vulnerable to signal jamming.

Landing on maritime vessels is another real-world application for UAVs that has gained popularity. Compared with landing on a moving ground carrier, landing on a moving shipdeck faces instability and complex motions resulting from ocean environmental disturbances, such as wind, gusts, turbulence from the ship’s airwake, and the ship–wave interaction due to a high sea-state. Additionally, sea-based landing makes conventional vision systems suffer from various illumination differences caused by sun reflections, rain, fog, and overcast skies, where some of the situations are considered the closest to nighttime conditions. An early study of [36] followed the strategy of placing a visual marker on an autonomous surface vehicle (ASV) for the UAV’s visual sensor to determine the proper landing area using a learning saliency-based approach, while the relative pose is measured with the help of an ASV-mounted upward-looking camera to capture a UAV-carried ArUco marker at close range before the final touch-down. Nonetheless, the field experiments were only conducted in the daytime under favorable weather conditions. A paper written by Sanchez-Lopez et al. [37] introduced a vision-based decision-tree method for classifying the international landing marker on a mimicked moving shipboard. The decision tree involves a combination of an artificial neural network and other geometrical properties of the landing marker, but it was only tested in laboratory environments as the heavy computational burden prevents the vision system from being implemented in real-time flight tests. Wang and Bai [38] proposed a visually guided two-stage process to land a quadrotor UAV on a moving deck simulator. The authors scaled and modified the landing pad with active LEDs to suit the onboard vision system in long-range and above-the-deck hovering for landing marker detection. For long-range detection, the onboard camera utilizes color segmentation to detect the red LEDs, whereas at close range, the conventional visual approach is adopted to detect the marker. Notice that the precision of the vision system was validated via VICON ground truth by an emulated deck motion under sea-state 6, but the average distance error of landing was relatively large compared with the size of the landing pad. The work of Xu et al. [39] customized a nested AprilTag on a moving vessel as the landing marker to assist UAV autonomous landing, in which the hierarchical marker design along with a three-stage marker detection scheme may suit the vision system at different altitude levels. Although a successful landing attempt was reported during the field tests, more details, such as the accuracy of the estimated relative pose and the speed of the moving vessel, were yet to be revealed. In our previous paper [14], we proposed a real-time vision system to successfully detect the international landing marker in a cluttered shipboard environment even in the presence of marker occlusion, of which closed-loop flight tests demonstrated the results.

Apart from the passive landing marker methods listed above, the active sourced markers presented in some early studies are potential solutions to nighttime landing. Activated landing markers, a large number of which are based on TIR technology that has been widely used for rescue, surveillance, inspection, and automatic target recognition [40,41], are more favorable to operating in bad weather conditions or dark environments. Several studies have suggested deploying infrared-based ground structures and sensors to accommodate the task of landing in poor-illuminated scenarios. For instance, Xu et al. [42] imported a “T”-shaped cooperative target emitting infrared light to couple with a long-wavelength infrared imaging sensor as the vision-based approach. Owing to the distinct advantage of infrared imaging, only affine moment invariants are processed for landing marker detection. Such a system has been validated under low visibility conditions, such as heavy fog, haze, and at night, showing a certain degree of accuracy and robustness. In [43], the authors constructed two concentric active IR markers to achieve robust UAV landing on a moving ground robot. The authors then simplified the vision algorithm to trivial brightness thresholding by mounting an IR-passing filter to the infrared camera to obscure all the visible light except the IR markers. Chen, Phang, and Chen [12] adopted a modified AprilTag illuminated with IR LEDs as the landing platform to allow tracking of a moving target in low-illumination conditions using the standard AprilTag recognition algorithm. Although the accuracy of the presented method has been validated, it lacks integration of the system to perform a fully autonomous landing. Similar infrared-based methods may also be found in [13,38,44]. These active-sourced markers have shown preferable performance against environmental limitations, including low illumination and bad weather conditions. However, we would not encourage their use due to the potential risks of exposing both the UAV and the landing site where stealthy operations are desired. Alternatively, it is of great necessity to achieve autonomous nighttime landing on a conventional landing marker due to the inevitable and increasing needs of UAV nighttime operations. To date, none of the existing approaches has demonstrated the ability to utilize autonomous landings at nighttime, counting on conventional vision sensors only.

## 3. System Configuration

The experimental system involved in this work has two major components: the unmanned aerial vehicle and ground-based facilities. The UAV carries essential avionics and computational equipment with the corresponding software to fulfill the requirements of onboard processing. At the same time, the ground-based facilities are comprised of a landing marker, a ground control station (GCS) laptop which enables accessing and monitoring the UAV remotely, and a router for establishing a wireless communication link between the UAV and the GSC.

### 3.1. The UAV Platform

We select a DJI Matrice 100 (M100) quadrotor equipped with a DJI E800 propulsion system as the UAV platform. It is a commercially off-the-shelf, fully integrated, highly flexible, and programmable aerial platform offering ready-to-fly capability and expandability. The quadrotor is able to perform GPS-guided waypoint navigation, GPS/attitude-based hover, and external-program-driven maneuvering. Such a setup minimizes the effort of customizing the airborne platform while enabling a more convenient and minor risky route toward actual flight tests. The M100 quadrotor has a maximum take-off weight and payload endurance of 3.6 kg and 1.0 kg, respectively. When equipped with a DJI TB48D 5700 mAh Li-Po battery, it guarantees a 20 min flight-time to cover most of the experimental tasks. Onboard avionics are involved, including an N1 flight control unit (FCU), an IMU consisting of onboard sensors such as gyroscopes, accelerometers, a magnetometer, and a pressure sensor, as well as a global navigation satellite system (GPS/BeiDou/GLONASS). A bi-directional serial interface running at a baud-rate of 921,600 bps enables the FCU to output IMU and flight states not limited to orientations, accelerations, velocities, and altitudes up to 100 Hz. External control commands can also be transmitted back to the FCU at a maximum rate of 100 Hz via the interface. A rechargeable RC transmitter has an operating range of up to 2 km, where the vehicle status, battery health, and homing position can be monitored when an external mobile device is attached.

**Figure 1:**
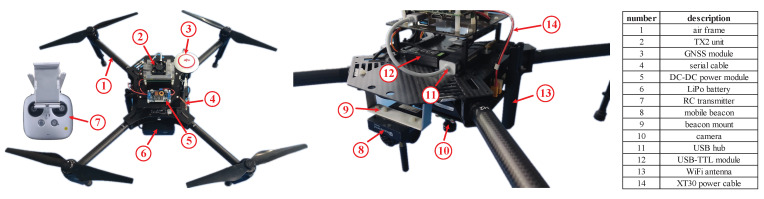
The DJI M100 quadrotor UAV platform and the customized payloads.

To fulfill the requirements of onboard processing, we customize some payloads and install them on M100. The payloads include an NVIDIA Jetson TX2 high-performance processing unit, a DC-DC power module, a CamNurse SY003HD 1080P color camera, a mobile beacon of the positioning system, and other custom-built mechanical structures for mounting purposes. The TX2 unit is equipped with a 256-core NVIDIA Pascal GPU, a dual-core NVIDIA Denver 2 64-Bit CPU, a quad-core ARM Cortex-A57 MPCore, 8 GB LPDDR4 memory, 32 GB internal storage, and multiple interfaces. It delivers 1.3TOPs performance under 15 W power consumption, which has become one of the most popular processing units for robotic applications. The DC-DC power module sources power from the TB48D LiPo battery and converts the voltage to suit the TX2 unit. The camera contains a 1/2.7 inch CMOS sensor offering a maximum resolution of 1920 × 1080, and a lens with focal length: 3.6 mm, FOV: 140${}^{{}^{\circ}}$ horizontal, f-number: 2.0, and maximum frame-rate: 30 Hz. The camera is tightened to a lightweight custom-built mechanism underneath the M100 battery cartridge facing straight downward and connected to the TX2 unit via a USB cable for target detection purposes. In the field tests, the camera operates at free-run mode with a resolution of 1280 × 1024 and a frame rate of 20 Hz. Figure 1 illustrates the M100 UAV platform and the customized payloads. Specifically, component No. 8 is a mobile beacon of a ground-based positioning system installed on the UAV’s airframe. We omit the detail here as it does not contribute to this work, whereas it is reserved for outdoor position referencing purposes in the ongoing studies.

### 3.2. Ground Facilities and Software Architecture

Apart from the UAV platform, ground-based landing facilities play an essential role in this research. A scaled landing marker is adopted to provide visual clues for landing guidance, details of which are depicted in Section 5. A wireless router powered by a LiPo battery has also been set up to bridge the M100 UAV and the GCS laptop so that the UAV onboard commands can be executed remotely by the GCS laptop via the Secure Shell Protocol (SSH).

The TX2 unit has an Ubuntu 16.04 Linux operating system and the robot operating system (ROS) kinetic version installed. ROS is an open-source embedded robot library designed to provide a standard for robotics software development, which abstracts robotic hardware from the software that any robot can use the codes. It is a distributed framework of processes that enables executables to be individually designed and loosely coupled at runtime. These processes are written in C++ or Python programming languages and often grouped in the form of packages, which can be shared, distributed, and reused conveniently. There is a variety of driver packages and third-party libraries that can be easily deployed and integrated. The major ROS packages involved in this work are the OpenCV library for visual algorithm implementation and the DJI Onboard-SDK for M100 sensor telemetry and aircraft condition monitoring. Additionally, ROS’ data recording, playback, and offline visualization functionalities are extensively used in experiments.

## 4. Low-Illumination Image Enhancement

Images acquired at nighttime by the onboard camera have extremely low illumination and visibility. Thus, the images need to be enhanced first before any follow-up processing can be applied. The solution to low-illumination image enhancement is twofold: increasing the exposure time to raise global brightness, and implementing specific image enhancement techniques. Unfortunately, the exposure time has an upper limit of 50 ms based on field experiments to guarantee a frame rate of 20 Hz to meet the requirements of real-time processing and hardware-in-the-loop control. Such an exposure time setup results in a very dark image in which details are hard to distinguish with the naked eyes despite partial artificial scene illumination existing. Therefore, a real-time image enhancement technique has been adopted to improve the quality of the images.

**Figure 2:**
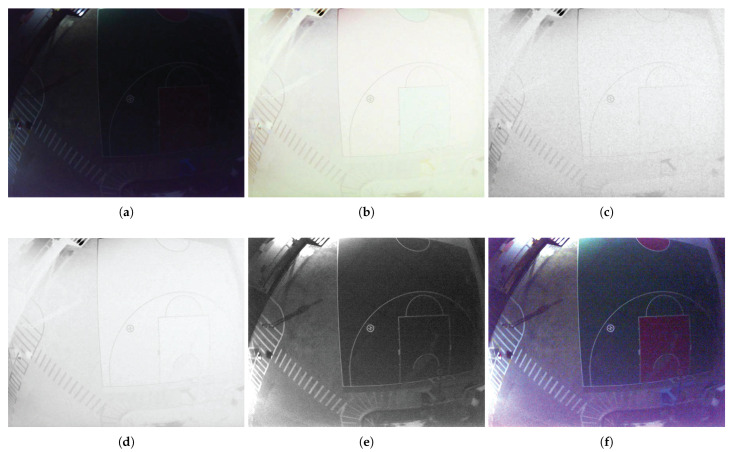
Low-illumination image enhancement: (**a**) Onboard captured low-illumination image. (**b**) Inverted low light image. (**c**) Result of dark channel calculation. (**d**) Result of luminance channel calculation. (**e**) Grayscale image after enhancement. (**f**) Color image after enhancement.

Conventional methods, such as gamma correction and histogram equalization, have shown adequate yet limited performance in low-illumination image enhancement. These methods are hindered by non-uniform brightness cases and suffer from over-enhancement of contrast, loss of details, and amplified background noise. In this section, a physical-model-based method for enhancing onboard capture image sequences in extremely low light conditions is presented. It treats image enhancement as haze removal, where the “haze” is determined by scene illumination. The proposed method is inspired by the work of Dong et al. [45]. Here, we adopt a similar but more efficient approach to facilitate low-illumination image enhancement. A channel-wise inverted low-illumination image has a very similar appearance to a hazy image:

(1) $$I_{inv}^{c}\left(x\right)=255-I^{c}\left(x\right)$$

where $I^{c}\left(x\right)$ is the input low-illumination image, $I_{inv}^{c}\left(x\right)$ is the inverted pseudo-hazy image, and *c* stands for the RGB color channels. Figure 2a,b show the low-illumination and inverted images of the test field, respectively. The McCarney atmospheric model is commonly adopted to describe the scattering process in hazy images:

(2) $$I_{inv}^{c}\left(x\right)=J^{c}\left(x\right)t\left(x\right)+A^{c}\left(1-t(x)\right)$$

in which $J_{inv}^{c}$ is the haze-free image yet to be estimated, $A^{c}$ is the global atmospheric light obtained by statistics, and t(x) is the transmission map describing the portion of the light reflected by the object and which reaches the camera. According to Dark Channel Prior (DCP) proposed in [46], a haze-free image has at least one color channel with very low intensity at some pixels:

(3) $$J^{dark}\left(x\right)=\underset{y\in \Omega (x)}{min}\left(\underset{c\in r,g,b}{min}J^{c}\left(y\right)\right)$$

where $J^{dark}\left(x\right)$ denotes the dark channel of image *J*, Ω(x) is a local patch centered at *x*. By taking the minimum operation in the local patch and performing dark channel calculation of the hazy image, Equation (2) can be re-written in the form of:

(4) $$\underset{y\in \Omega (x)}{min}\left(\underset{c\in r,g,b}{min}\left(\frac{I_{inv}^{c}\left(y\right)}{A^{c}}\right)\right)=\underset{y\in \Omega (x)}{min}\left(\underset{c\in r,g,b}{min}\left(\frac{J^{c}\left(y\right)}{A^{c}}\right)\right)\tilde{t}\left(x\right)+\left(1-\tilde{t}\left(x\right)\right)$$

The transmission in a local patch Ω(x) is assumed to be a constant, and the patch’s transmission is denoted by $\tilde{t}\left(x\right)$. According to DCP, $J^{dark}\left(x\right)$ in Equation (3) is assumed to be zero, as $A^{c}$ is always positive. Combining Equations (3) and (4), a coarse transmission map $\tilde{t}\left(x\right)$ is derived with a constant parameter ω to retain a small amount of haze for depth perception:

(5) $$\tilde{t}\left(x\right)=1-\omega \underset{c}{min}\left(\underset{y\in \Omega (x)}{min}\left(\frac{I_{inv}^{c}\left(y\right)}{A^{c}}\right)\right)$$

Then, the haze-free image J(x) can be recovered according to Equation (6), where $t_{0}$ is a lower bound to preserve a small amount of haze in dense hazy regions. By inverting J(x) again, the enhanced image is obtained.

(6) $$J^{c}\left(x\right)=\frac{I_{inv}^{c}-A^{c}}{max\left(t\left(x\right),t_{0}\right)}$$

(7) $$J_{enh}^{c}\left(x\right)=255-J^{c}\left(x\right)$$

Although the original DCP method can enhance low-illumination images, it is subject to several drawbacks. One of the disadvantages is that the patch-based calculation in the estimation of $\tilde{t}\left(x\right)$ consumes a significant amount of computational power while introducing block artifacts in the transmission map, as the transmission is not always constant in a patch. Another problem is that the density of haze is caused by the intensity of light rather than scene depth, yet such a fact is ignored in the DCP model. Therefore, we relax the restrictions of patch-based calculation and use the luminance channel to substitute the dark channel. Figure 2c,d are the corresponding dark channel and luminance channel images derived from Figure 2b, respectively. We may observe that the luminance channel image is superior to the dark channel one due to the suppressed speckle noise. A simple grayscale conversion obtains the luminance channel image:

(8) $$I_{inv}^{l}\left(x\right)=0.299\times I_{inv}^{r}\left(x\right)+0.587\times I_{inv}^{g}\left(x\right)+0.114\times I_{inv}^{b}\left(x\right)$$

We also discover that the pixels of the transmission map $\tilde{t}\left(x\right)$ calculated by DCP and the pixels of $I_{inv}^{l}\left(x\right)$ are roughly symmetrical about the line y=k. As presented in Equation (9), *k* is a tunable constant that dominates the effect of enhancement. The improper value selection of *k* leads to over-enhancement or insufficient enhancement. Therefore, *k* should be chosen carefully.

(9) $$\tilde{t}\left(x\right)-k=-\left(\frac{I_{inv}^{l}\left(x\right)}{A^{l}}-k\right)$$

Hence, combining Equations (6) and (9), we may utilize a simplified method to obtain a haze-free image using Equation (10). The value of *k* has been empirically set to 0.52. Such a value achieves the best result while preventing over-enhancement. For estimating the global atmospheric light $A^{l}$, the original DCP method has an extra statistical step because the brightest pixel in the image does not always represent the atmospheric light. Conversely, we relax this assumption and pick the brightest pixel in the luminance channel image $I_{inv}^{l}\left(x\right)$ for $A^{l}$. The reason behind this is that the intensity of the pixels in image $I_{inv}^{l}\left(x\right)$ is only related to the level of darkness in the original low-illumination image. As $I_{inv}^{l}\left(x\right)$ is an inverted image, the brightest pixel of $I_{inv}^{l}\left(x\right)$ always represents the darkest spot of the scene that needs most enhancement. To speed up the calculation, we only process the luminance channel image to reduce the computational burden and prevent further yet redundant color-to-grayscale conversion. This is because the landing marker adopted in this work is of bright color on a dark background that can be easily distinguished in a grayscale image, whose details are elaborated in the next section. After haze removal, the inverted image after enhancement $I^{l}\left(x\right)$ is shown in Figure 2e. We also present the color version of the enhanced image in Figure 2f for visualization and an intuitive comparison.

(10) $$\hat{J^{l}}\left(x\right)=\frac{I_{inv}^{l}-A^{l}}{max\left(2k-I_{inv}^{l}\left(x\right),t_{0}\right)}$$

## 5. Landing Marker Localization

The main objective of landing marker localization is to robustly detect the landing marker and extract its relative pose with respect to the UAV. In this section, a novel hierarchical approach is presented to deal with landing marker localization after low illumination image enhancement. It includes a hierarchical scheme consisting of a pre-processing stage to reduce noise and separate the candidate foreground, a decision tree in connection with a lightweight CNN for detection and validate the landing marker, and an information extraction method to obtain the critical relative pose information for target tracking and closed-loop landing control. The approach minimizes the complexity of the system to achieve real-time processing while maintaining detection accuracy and robustness.

### 5.1. Landing Marker Analysis

Marker-based approaches have been commonly involved in UAV landing. Since increasing the versatility becomes more of a necessity, we aim to use a general marker, the international landing pattern consisting of a block letter “H” and a surrounding circle, to extend the adaptability of landing. Whereas some other marker-based methods have specific requirements on the combination and size of the visual patterns, our visual approach does not rely on a special configuration of marker size or proportion. Instead, the width, height, and thickness of the block letter and the circle can be freely customized and adjusted in practice.

**Figure 3:**
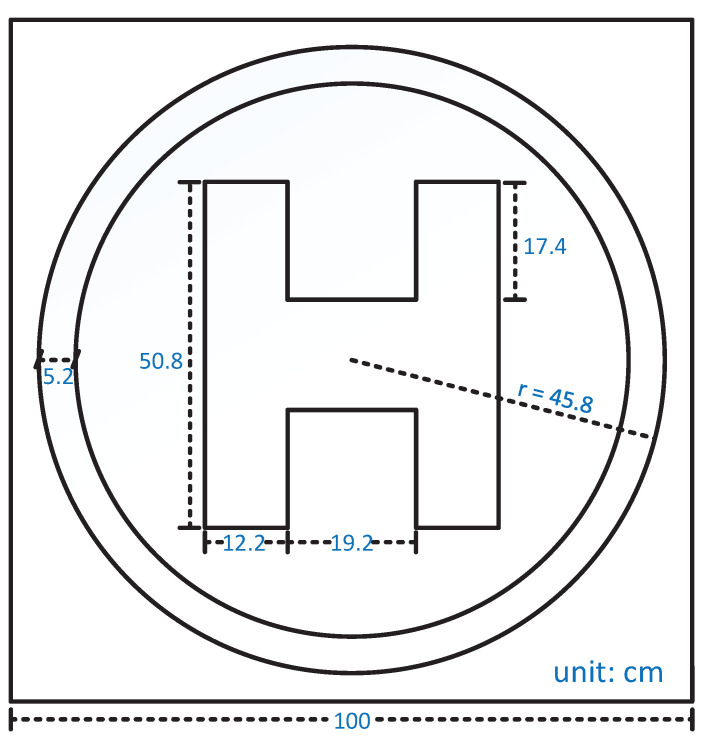
Details of the scaled international landing marker.

As illustrated in Figure 3, a scaled landing marker of size 1 × 1 m${}^{2}$ is adopted in our work, in which the circular pattern has a radius of 45.8 cm, and the block letter “H” has a width of 43.6 cm and a height of 50.8 cm. The landing marker is of bright color and painted in a gray background to be sufficiently distinguishable. According to the lens setup, the marker is also adequately large for the onboard vision sensor to see it at an altitude up to 15 m. It is worth mentioning that no further modification has been applied to the landing marker, as we would like the visual approach developed in this work to be adaptable to other scenarios where a similar landing marker is presented.

### 5.2. Hierarchical-Based Marker Detection and Validation

#### 5.2.1. Hierarchical Mission Definition

As the UAV moves towards the landing marker from long distances to finally land on it, one of the significant challenges is the scale change of the marker in the image. At high altitudes, the marker becomes relatively small so that certain features are too difficult to be detected, but at the same time, the vehicle is less likely to encounter obstacles. At low altitudes, the situation is just the opposite. Therefore, we employ a hierarchical-based scheme to divide the marker detection problem into four different phases, named “Approaching”, “Hovering”, “Descending”, and “Touch Down”, according to a variable Δ for altitude. In this study, Δ was empirically set to 1 m based on the marker size and experiments. We utilize the UAV’s onboard GNSS module to receive altitude information, so that the vision system is able to determine the mission phase. The details of the hierarchical-based scheme are depicted as follows:

- At the “Approaching” phase with an altitude level above 8Δ, the landing marker is acquired at a distance and forms a small area in the camera image. A piece of coarse position information is sufficient to guide the vehicle towards the marker. Hence, a rough estimation of the marker center with an acceptable position error is all we need at this stage. We also affirm that 15Δ is the maximum altitude to detect the marker with considerable accuracy, whereas the accuracy of the visual algorithm rapidly degrades when the altitude exceeds the limit.
- The second phase is defined as the “Hovering” phase. When the UAV enters an altitude interval between 3Δ to 8Δ, it hovers and continuously tracks the marker to minimize the position error. Therefore, a more precise position estimation is required. The landing marker offers more visual detail for hover and altitude descending control than the “Approaching” stage.
- During the third phase, “Descending”, the vehicle lowers its altitude to an interval between 1Δ to 3Δ and prepares for the final landing stage. During this process, the relative pose between the UAV and the marker is derived using all the critical visual clues of the marker for landing control.
- Finally, the UAV enters the “Touch Down” phase when the altitude is below 1Δ. At this time, the onboard camera is too close to the ground, so that the landing marker is very likely to fade out and no longer present itself. The vehicle relies on the rest of the onboard avionics, as well as the state machine, to predict the relative pose to accomplish touch-down control.

#### 5.2.2. Pre-Processing

**Figure 4:**
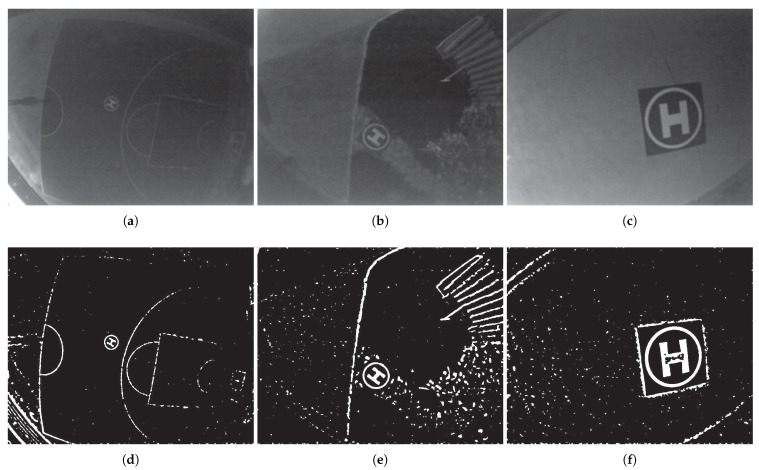
Adaptive thresholding results at different scenes and altitudes: (**a**–**c**) The enhanced and filtered field experiment images captured at the “Approaching” phase (GNSS altitude 8.32 m), the “Hovering” phase (GNSS altitude 6.58 m), and the “Descending” phase (GNSS altitude 2.91 m), respectively. (**d**–**f**) The corresponding images after adaptive thresholding using box sizes 29, 41, and 51, respectively.

We observe that a nighttime image is corrupted by shot noise with a mixture of Poisson-Gaussian distribution due to the inherent drawback of the image sensor. Such noise is significantly amplified after low-illumination enhancement, which affects image binarization and landing marker separation. The first step of marker detection is to reduce noise and isolate the foreground objects. Noise reduction is performed by employing a 5×5 mean filter along with a 7×7 Gaussian filter, resulting in a slightly blurred image. Due to the uneven distribution of scene luminance, adaptive thresholding is applied to binarize the image to capture the border of the landing marker. Different box sizes are selected according to altitude levels, that is: box size 29 for altitude above 8Δ, 41 for altitude between 3Δ to 8Δ, and 51 for 1Δ to 3Δ. We suggest that such a box size configuration maintains a balance between processing speed and quality. The input images and the corresponding results after adaptive thresholding can be seen in Figure 4a–f.

After thresholding, connected component analysis is performed to select the candidate region of interest (ROI) for further validation. Since there are many small irregular blocks in the binarized image, we apply different minimum area thresholds to the generated connected components based on the hierarchical proposal to bypass the irrelevant blocks. We set thresholds to 150 pixels for the “Approaching” phase, 500 pixels for the “Hovering” phase, and 1200 pixels for the “Descending” phase, respectively, based on an image resolution of 1280×1024. Such a scheme effectively reduces the number of connected component candidates from thousands to less than a dozen per frame on average. Then, the minimum bounding box is computed for each remaining connected component to form an ROI in both the previous enhanced image and the binarized image. Then, the results are forwarded to a decision tree for marker detection and validation.

#### 5.2.3. Decision Tree Validation

The decision tree presented in this work contains four nodes in total. The first node is to check if the shape of the ROI is similar to a square due to the landing marker being asymmetric. Since perspective and lens distortions may change the ratio, a tolerance $\delta_{1}$ is applied to such a criterion to improve the robustness. The second decision node is based on the ratio between the number of pixels from the outer circular pattern, and that of the “H” pattern is close to a constant value $c_{1}$, while the ratio between the number of pixels from the entire pattern and the dark background is close to a constant value $c_{2}$. Taking the factors of occlusion and perspective distortion into account, tolerances $\delta_{2}$ and $\delta_{3}$ are applied to $c_{1}$ and $c_{2}$ to improve the robustness. Such a feature is scale-, translation-, and rotation-invariant. The confirmed ROIs are sent to the next node. The third node is to check whether a binarized ROI image contains candidate graphical components or not. It examines if the connected components from the candidate ROI have nested holes to determine whether a complete landing marker is presented or not. We also take the situation of occlusion into account. If partial occlusion contaminates the marker, the circular pattern is likely to be incomplete, which violates the nested-holes assumption. Instead, if at least two connected components are within one single ROI, and the geometrical centers are within a certain pixel range, the ROI is confirmed as a potential candidate and passed on to the next tree node. The criteria mentioned above are also scale-, translation-, rotation-invariant, and anti-occlusion features. ROIs that do not meet the criteria are discarded.

**Figure 5:**
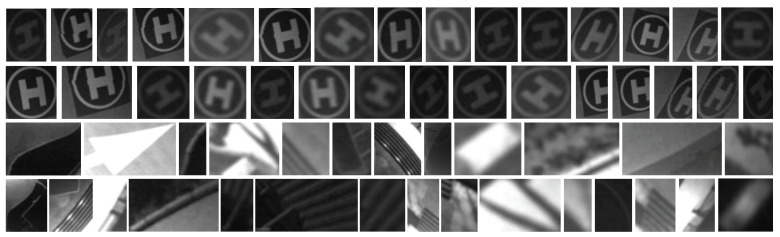
Selected images from the training dataset.

The fourth node is the most critical yet resource-consuming node, in which a CNN is developed to perform marker validation. Unlike the region-proposal-based or bounding-box-regression-based CNN frameworks, we treat the landing marker detection problem as a simple binary classification problem using the ROIs extracted from the previous node. As a result, our method only validates the extracted ROI images using a lightweight network architecture rather than searching for the desired target in the entire image. Such an approach significantly accelerates the processing speed 20 times faster than the state-of-the-art (SOTA) CNN object detectors. To generate the training samples, we first utilize the previous ROI images containing landing markers and segmented natural scenes of different scales, perspective angles, and illumination conditions captured under various scenarios to construct the dataset. In total, 100 landing marker images and 200 natural scene images are manually chosen as the training dataset, whilst another 20 landing marker images and 40 natural scene images are randomly chosen as the testing dataset. In Figure 5, the selected images of the dataset are illustrated, where the first two rows are the captured landing markers, and the last two rows are the segmented natural scenes. Notice that some markers are partially occluded, heavily distorted, blurred, and affected by discontinuity to improve the network’s generalization ability. Before training, each image is resized to a resolution of 56×56 and normalized to a range of [0, 1]. In order to further extend the adaptability of the network, data augmentation, including affine transformation, random cropping, and random rotation, is applied to the training samples.

**Figure 6:**
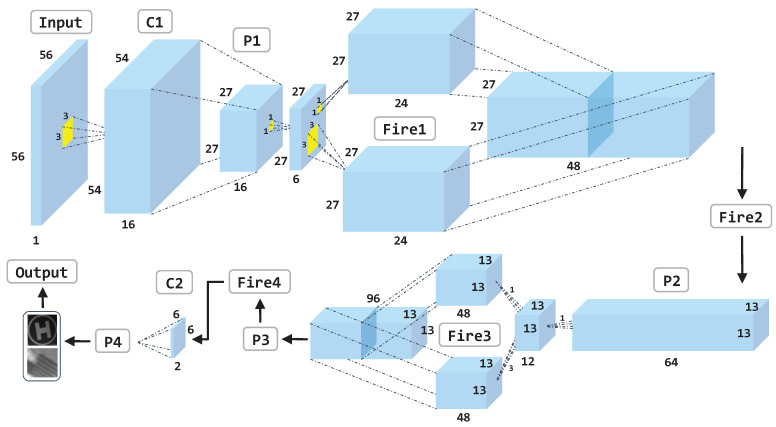
The proposed lightweight CNN architecture for marker validation.

As the developed CNN is eventually running on a resource-constrained UAV onboard platform, we are not encouraged to use deep network architectures such as ResNet [47] and VGG [48] to trade processing speed for precision. Instead, we present a simple yet efficient network structure primarily inspired by SqueezeNet [49] to facilitate landing marker validation. The key idea is to substitute the conventional convolution layers with the “Fire” modules to reduce computation and parameters. A fire module is comprised of a squeeze convolution layer with 1×1 filters only, followed by an expand layer consisting of two separate convolution layers: one with 1×1 filters, the other with 3×3 filters, outputs of which are concatenated together in the channel dimension. As depicted in Figure 6, the network begins with a standalone convolution layer C1 using an input image of size 56×56, followed by four fire modules Fire1 to Fire4, and ends with another standalone convolution layer C2. Max-pooling with stride 2 (P1 to P3) are employed after C1, Fire2, and Fire3, respectively. As the input of the proposed network is only a quarter of the original SqueezeNet, we proportionally reduce the number of filters per fire module, resulting in (6, 24, 24) for Fire1, (8, 32, 32) for Fire2, (12, 48, 48) for Fire3, and (16, 48, 48) for Fire4, respectively. A dropout layer with a ratio of 40% is applied after the Fire4 module. The final average pooling layer P4 divides the output into landing marker and background categories, where a softmax layer is used to classify the output. For training purposes, the network is trained by minimizing the cross-entropy loss function using back-propagation on a desktop PC equipped with an Intel i7 4790K CPU, 16GB DDR3-1333MHz RAM, and an Nvidia Geforce 1070 GPU running Ubuntu 16.04 OS. We used a batch size of 4, a learning rate of 0.001, and an Adam optimizer to train the network for 100 epochs under PyTorch framework.

#### 5.2.4. Keypoint Extraction

**Figure 7:**
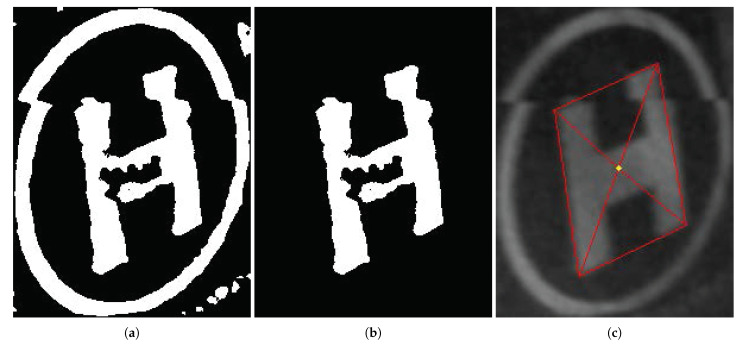
Landing marker keypoint extraction: (**a**) Small outliers are first rejected from the binarized ROI image. (**b**) The circular pattern is removed, and the “H” pattern is reserved. (**c**) The vertices and center are obtained by fitting a bounding parallelogram.

Finally, if an ROI is affirmed as a landing marker by the CNN node, we extract the keypoints from the corresponding binarized ROI image and preserve them for further processing, such as pose estimation. Specifically, we treat the four outermost vertices and the center of the “H” pattern as identical points. As illustrated in Figure 7a, the vision system first rejects the small outliers of the marker ROI image, then removes the circular pattern to obtain a clear image of the “H” pattern (see Figure 7b). The four vertices of the bounding parallelogram of the “H” pattern are extracted, and the marker center is derived by simply averaging the pixel coordinates of the vertices, as shown in Figure 7c.

## 6. Experiment and Discussion

### 6.1. Field Experiment Scenes

**Figure 8:**
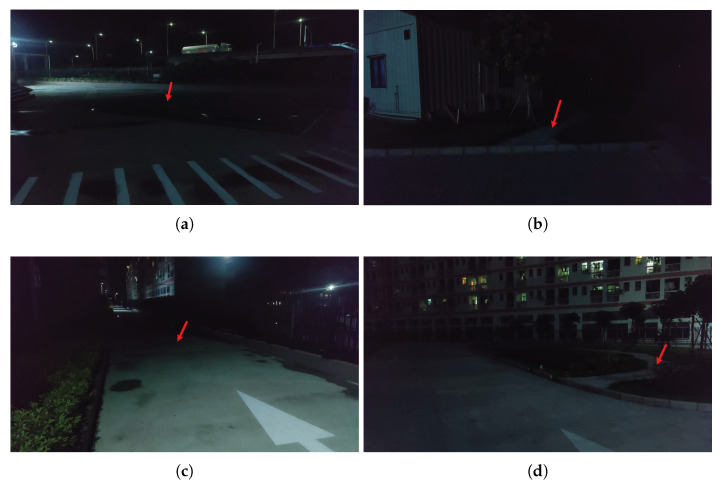
Selected scenes at the South China University of Technology—Zhuhai Institute of Modern Industrial Innovation campus for field experiments: (**a**) The basketball field. (**b**) The warehouse. (**c**) The fire escape. (**d**) The small garden. The red arrow in each figure indicates the landing marker’s actual position during the field tests with a measured average luminance of 5 lx.

Since this is the first attempt, we try to perform vision-based landing marker detection in low-illumination nighttime environments, and we conduct a comprehensive investigation to select the proper locations at the South China University of Technology—Zhuhai Institute of Modern Industrial Innovation campus before carrying out the field tests. The majority of the campus has intensive artificial light sources around buildings and paths. We have chosen four different scenes with minimum light sources but cluttered backgrounds as the experiment locations, as illustrated in Figure 8a–d. Additionally, the red arrow in each scene indicates the actual position where the landing marker is placed in the experiments. We also employ a light meter with a precision of 0.1 lx and a range of 200,000 lx to measure the luminance at each marker location.

To be more specific, Figure 8a is the basketball field of the campus near the main building with the nearest light source approximately 15 m away, shown in the top-left corner of the figure. The landing marker is placed in the middle of the field (denoted as L${}_{1}$), where the measured luminance is 4.0 lx. In Figure 8b, the landing marker is placed on a stone-paved path near a small warehouse (denoted as L${}_{2}$) and surrounded by some vegetation with a measured luminance of 4.3 lx. In Figure 8c, the landing marker is placed on the dark side of a fire escape (denoted as L${}_{3}$) with a luminance of 4.8 lx, while the luminance at the white arrow marking is approximately 32 lx due to closer to light source. In Figure 8d, the landing marker is placed in a small garden (denoted as L${}_{4}$) on a stone-paved path surrounding by some vegetation, where the measured luminance is 7.6 lx. The averaged luminance at the marker locations is approximately 5 lx. We conduct various field experiments at the above-mentioned scenes and record the onboard videos in the late evening at around 10:00–12:00 p.m. with moderate weather conditions.

### 6.2. Vision System Evaluation

**Figure 9:**
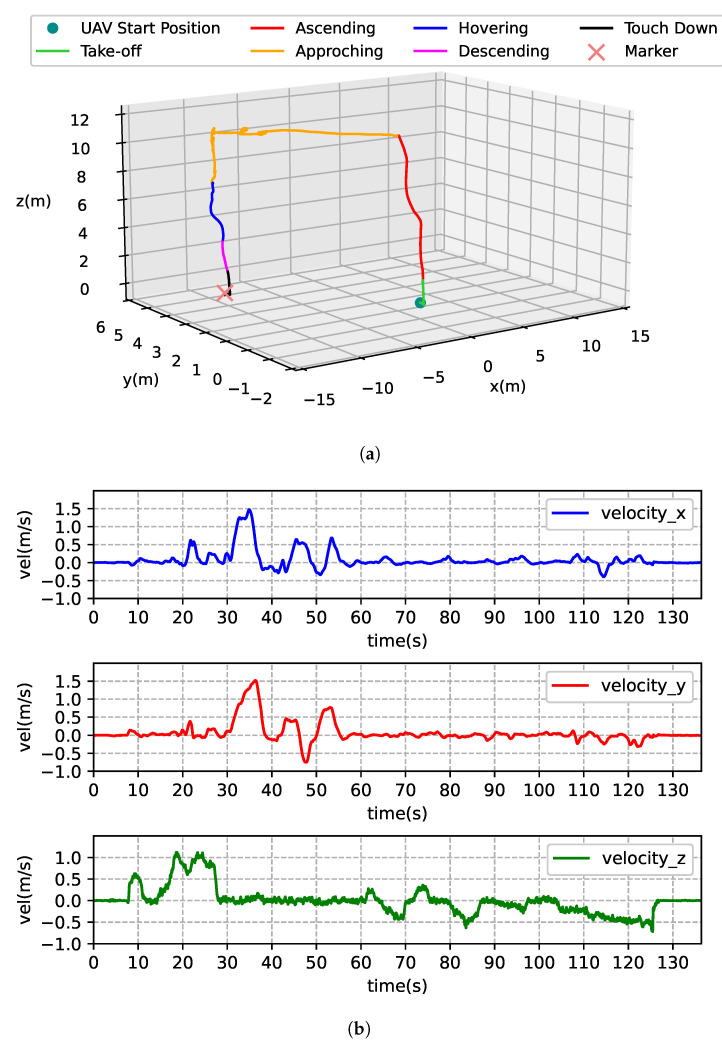
One of the recorded manual flight tests. (**a**) The three-dimensional plot of the quadrotor’s trajectory for mission phase determination. (**b**) The quadrotor’s local velocities of the *x*, *y*, and *z* axes.

To evaluate the vision system proposed in this paper, we follow the complete procedure of autonomous landing and manually fly the DJI M100 quadrotor to simulate the phases of “Take-off”, “Approaching”, “Hovering”, “Descending”, and “Touch Down”. In Figure 9a, the three-dimensional trajectory of one manual flight test recorded by the onboard GNSS module is presented. As we can see, the quadrotor takes off from the vicinity of the landing marker at the beginning. After ascending to an altitude above 10 m, it approaches the marker and stays in the air to confirm target detection. Then, the vehicle gradually lowers its altitude during the “Hovering” and “Descending” phases and eventually lands on the marker. Figure 9b shows the vehicle’s local velocities of the *x*, *y*, and *z* axes. The onboard camera records images at a resolution of 1280 × 1024, a frame rate of 20 Hz, and an exposure time of 50 ms. As mentioned earlier in Section 3 and Section 4, such a frame configuration meets the minimum requirement of real-time processing while allowing as much light as possible to enter the camera in the exposure phase to lighten the image. Each video is then stored in the “bag” file format under the ROS framework. The actual frame rate is around 19.8 Hz due to processing latency between adjacency frames, but we neglect the error in this study. In the experiments, the M100 quadrotor has a maximum horizontal speed of 2 m/s and a vertical speed of 1 m/s, which is sufficient to simulate motion blur and perspective distortion caused by varying the camera angle when acceleration and deceleration occur horizontally. Finally, at each field experiment scene, we collect a video with a length of approximately 2–3 min, resulting in a total number of four videos for evaluating the proposed vision system. In Table 1, the statistics of each video are summarized, where key elements like “total images”, “marker images”, and “max altitude” are presented. These elements are the fundamental metrics for evaluating the vision system.

Since our dataset is built on the automatically-extracted ROI images without precisely labeled ground-truth bounding boxes, it would be inappropriate to use the conventional intersection of union (IoU) metric to evaluate the vision system. Instead, we employ some other criteria to evaluate the vision system based on the following equations:

(11) $$\mathrm{TP}=\exists C_{m},\;if\;\left|C_{m}-C_{g}\right|<=r_{th}\;r_{th}=H_{d}/8$$

(12) $$P=\frac{\mathrm{TP}}{\mathrm{TP}+\mathrm{FP}}\;\;,\;R=\frac{\mathrm{TP}}{\mathrm{TP}+\mathrm{FN}}$$

(13) $$F_{1}=\frac{2\mathrm{PR}}{P+R}$$

where points $C_{m}$ and $C_{g}$ are the predicted landing marker center and its ground truth, respectively. Equation (11) indicates that if the marker center is successfully detected when a marker is presented, whose coordinate falls into a small circle *C* centered at $C_{g}$ with radius $r_{th}$, we consider the detected landing marker as a true-positive TP. According to our previous experience in [14], a marker center derived from the four outermost vertices of the “H” pattern is accurate enough with limited bias. Hence, we set $r_{th}$ to one-eighth of the bounding polygon’s diagonal length. Such a value raises the threshold and makes the evaluation more reasonable. On the contrary, if a landing marker is presented in the image, but no marker center is obtained, or the predicted marker center is outside the circle *C*, it is considered a false-negative FN. For the extracted background ROI images, if an image is categorized as a landing marker by the CNN node, it is considered a false-positive FP. In Equations (12) and (13), the standard metrics, precision P, recall R, and F-measure F${}_{1}$, are adopted to evaluate the accuracy of the vision system. Compared with the conventional IoU metric, the proposed criteria give credit to both the quality of landing marker detection outcomes and the detection rate. The overall performance of the vision system affects the accuracy of pose estimation and precision of hardware-in-the-loop control in future works.

Based on the criteria mentioned above, we collect the output images of each stage using the recorded different scene videos, and manually categorize them into marker (denoted as “MK”) and background (denoted as “BG”) categories to calculate the exact numbers of TP, FP, and FN, results of which are listed in Table 2. Specifically, the term “ccomp” stands for the number of detected connected components, and “dt1-dt3” means the remaining ROIs filtered by nodes 1 to 3 of the decision tree. Owing to the CNN node playing a critical role in confirming actual landing markers and rejecting the irrelevant background ROIs, the output of the CNN node is counted separately and presented as “dt4_cnn”. Finally, we examine the number of cases where the marker keypoints can be successfully extracted, denoted by the term “ext_kpt”. At scene L${}_{1}$, there are 2380 marker-presented frames, whereas the “ccomp” stage outputs 2339 connected components of the marker and 5186 that of background fragments. Then, the decision tree rejects a small amount of the marker ROIs and outputs 2241 marker images with accurately extracted keypoints confirmed as TP while effectively eliminating more than 98% of the background segments. We carefully examine the remaining FP samples reported by the network, discovering that they all come from partially cropped landing marker images generated during the final “Touch Down” phase of landing. Although the keypoints are drawn from most of these “H” patterns, we still consider them FP due to inconsistency and unreliability. Compared with L${}_{1}$, scene L${}_{2}$ has a more complicated and cluttered background than the basketball field. Therefore, nearly three times the number of the connected components more than L${}_{1}$ are picked up in L${}_{2}$. The vision system has similar performance to the previous scene, except that the CNN node only verifies 23 FP samples. We study this case and find the cause lies in the way of the landing maneuvering. Since the marker is placed on a narrow stone-paved path surrounded by cluttered vegetation, the quadrotor is piloted toward the west of the marker at a relatively high altitude (3.28 m) to land on flat ground. The marker quickly vanishes by the edge of the image, leaving limited partially occluded marker samples only. However, we find it difficult to extract the keypoints from these negative samples due to heavy motion blur. Moving on to the fire escape scene L${}_{3}$, an interesting phenomenon is observed that the number of extracted markers is slightly greater than its actual value (3008 vs. 2936, marked by the ${}^{*}$ sign in Table 2, column 6). This is because the fire escape has a lighter color background than the marker. The edges of the marker square are thresholded to the foreground at a low altitude, leading to approximately 80 repetitive identifications of the marker. Therefore, we have subtracted this number at each stage to make a fair comparison. Scene L${}_{4}$ has a similar cluttered environment as scene L${}_{2}$, where a large number of background segments have been successfully rejected by the vision system while achieving a comparable performance in marker detection. A majority of the FP samples also come from incomplete marker patterns that are yet to be eliminated.

In Table 3, precision P, recall R, and F-measure F${}_{1}$ are listed correspondingly. Combining with the altitude information listed in Table 1, we may also see that the altitude level has an impact on the system. The vision system occasionally misses the marker when the UAV flies above 13 m due to insufficient pixels in the connected component analysis. This situation is exacerbated if motion blur generated by horizontal movements occurs, which explains a slight degeneration in system performance found in scene L${}_{2}$. In contrast, the vision system achieves the best performance at scene L${}_{3}$ with an altitude constantly below 10 m. Note that we subtract the repetitive identifications mentioned above from final system outputs to get the actual number of TP samples (2852, denoted by ${}^{*}$ sign in Table 3) at scene L${}_{3}$. In real landing scenarios, it is recommended to maintain a lower altitude for landing marker detection and tracking.

**Figure 10:**
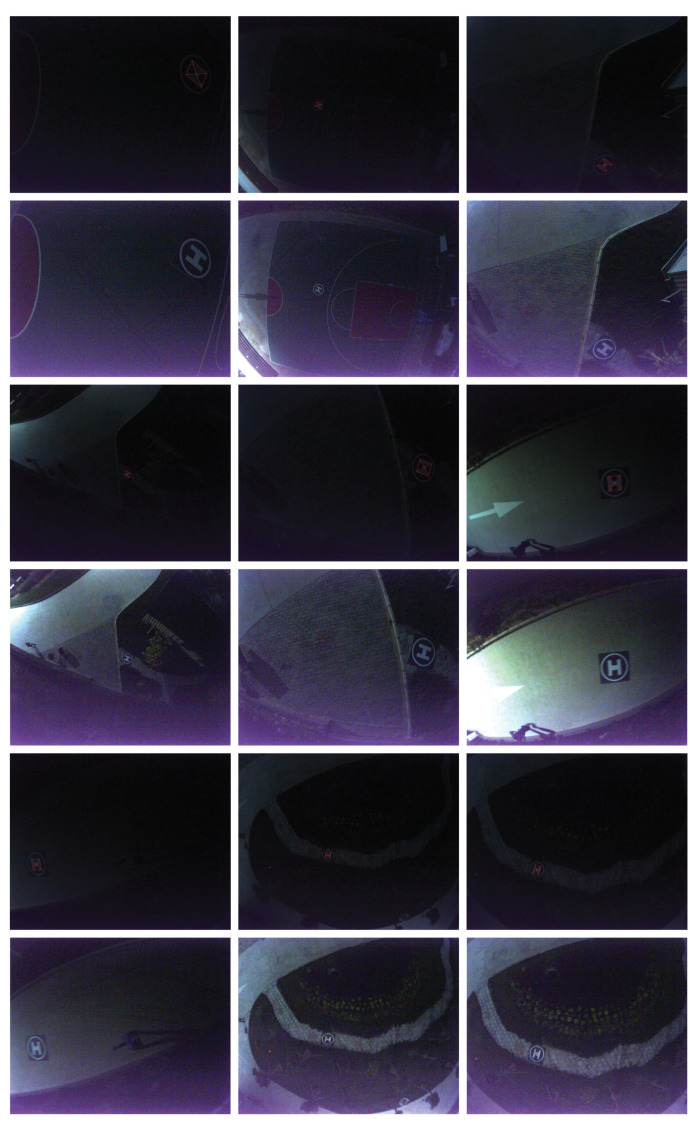
The landing marker detection results of the proposed vision system. The first, third, and fifth rows: Selected color images from the original test dataset. Detected markers are highlighted in color lines and dots. The second, fourth, and sixth rows: The corresponding color images after enhancement for scene detail observation.

In Figure 10, we present some of the landing marker detection results. The first, third, and fifth rows are some of the original color images captured onboard, in which the markers detected by our vision system are highlighted in color lines and dots. The second, fourth, and sixth rows are the corresponding color images after enhancement to observe the details. The proposed vision system is able to detect the landing marker in the presence of noise, scaling, image distortions, motion blur (see figures in row 4, column 3 and row 6, column 1), and image acquisition error (see the last two figures of Figure 10) in complex low-illumination environments.

As we suggest, the low-illumination image enhancement stage plays a crucial role in landing marker detection, and the vision system is further evaluated using the same metrics, but the original test videos are without enhancement. To make a fair comparison, we also retrain the CNN node with the original unenhanced training samples. The results are shown in Table 4, from which we may intuitively observe massive degradations in system performance. The reason is that the pre-processing stage of the system experiences difficulty properly binarizing the image, thus reducing the number of generated connected components and detection rates in the subsequent stages. Notice that only two marker samples can be identified at scene L${}_{1}$, leading to a complete failure of the system. Such a bad performance is because the marker is placed at the basketball field’s center, a location far away from the light source with the lowest luminance among all the test scenes. The landing marker merges into the dark background so that it can no longer be detected. According to the analysis, we may conclude that the low-illumination image enhancement scheme increases the quality of nighttime images and is also the foundation of landing marker detection.

Since there is a certain number of CNN-based object detection frameworks in the related research, we also compare the performance of the proposed vision system against that of the state-of-the-art methods that can be implemented in real-time. We have chosen YOLOv3 and its simplified version YOlOv3-Tiny [50], and MobileNetV2-SSD [51] to carry out the evaluation. As our vision system does not rely on image-wise bounding box labeling, we randomly select an equal proportion of the original images without enhancement from the videos to establish a dataset for a fair comparison. From Table 1, one may see there is a total number of 9642 marker images. As a result, 500 images were selected and manually labeled as the training dataset, whilst the remaining 9142 images were collected for testing. All the images were resized to 640×512 for a faster evaluation process.

Each network was trained individually using the default parameters under official guidance, and the detection results are listed in Table 5. It is worth mentioning that the M100 quadrotor spends a longer time at higher altitudes during the “Approaching” and “Hovering” stages, resulting in approximately 60% to 70% of the images appearing to have a relatively small landing marker, which brings difficulties to marker detection. As a result, it significantly increases the number of FN samples for YOLOv3 and its derivative. On the contrary, MobileNetv2-SSD performs better than YOLOv3 and YOLOv3-Tiny in detecting small markers, but also introduces a certain amount of FP samples, which are other ground objects, into the results. Finally, the performance of our vision system is derived by using the data from Table 3, from which one may see that our approach achieves the best recall and F-measure based on a minimal network design.

We also evaluate whether the enhanced low-illumination images benefit the above-mentioned CNN-based object detection frameworks or not. Again, each network is trained and tested by the same procedure, but using the enhanced image dataset. We present the detection results in Table 6. We may see some overall improvements for both YOLOv3 and YOLOv3-Tiny, whereas MobileNetv2-SSD significantly reduces the FN samples to achieve the best recall and F-measure. Note that such a result slightly outperforms our vision system, as the enhanced images offer much stronger and more discriminative features for the networks to extract. However, our vision system still has the advantage of processing speed, which are elaborated in the next subsection.

### 6.3. Processing Time Evaluation

Processing speed is of crucial importance to real-time UAV applications. Therefore, the timing performance of each stage of the proposed system is quantitatively evaluated. In this section, we utilize the collected videos to test the average time consumption of each processing stage. Each video has an initial resolution of 1280 × 1024, that is then resized to 1024 × 768 and 800 × 600, respectively. We use the annotations “HR”, “MR”, and “LR” to denote these resolutions. The tests are conducted on both the CNN training desktop PC (denoted by “PC”) and the NVidia TX2 unit (denoted by “TX2”). Timing performances of different processing stages, including image enhancement, pre-processing, the decision tree method, and keypoint extraction are comprehensively evaluated, results of which are listed in Table 7 accordingly. It is worth mentioning that the CNN node calculation is performed on GPUs on both the platforms, where the time consumption of data transfer between CPU and GPU is neglected.

We may see that the most time-consuming parts lie in the image enhancement and the adaptive thresholding stages. By reducing the resolution to the “MR” level, the detection rate on the desktop PC has surpassed the maximum frame rate that the onboard camera offers. On the contrary, the TX2 unit has relatively poor performances on both the “HR” and “MR” resolutions. Nonetheless, it has already achieved a detection rate of more than 10Hz on the “LR” resolution, satisfying this study’s minimum requirement of real-time processing. Compared with the works of [9,25], we utilize the landing marker detection at a higher resolution, whereas their algorithms only operate at image resolutions of 752 × 480 and 640 × 480, respectively. We also test the timing performance of the aforementioned CNN-based frameworks on the desktop PC’s NVidia Geforce 1070 GPU. For an image size of 1280 × 1024, the averaged processing time for YOLOv3, YOLOv3-Tiny, and MobileNetV2-SSD are 102.65 ms, 14.12 ms, and 64.39 ms. For image size 640 × 512, YOLOv3, YOLOv3-Tiny, and MobileNetV2-SSD take approximately 33.49 ms, 5.21 ms, and 26.65 ms to process one frame. Although these networks may achieve real-time processing using a high-performance desktop GPU at the cost of reducing the image resolution, we still find it challenging to implement these networks on a resource-limited UAV onboard platform. Moreover, these networks still require the low-illumination image enhancement scheme to achieve a better performance, which further reduces the processing speed.

Note that the timing performance of the proposed vision system is accomplished without optimization, in which redundant operations are executed at each frame. Using the information of adjacent frames in the image sequences, we may narrow down the ROIs to a specific area based on the previous detection results to dramatically reduce the computational burden caused by image enhancement and adaptive thresholding.

## 7. Conclusions and Future Works

In this paper, we presented a novel, robust, and efficient vision system for assisting UAV autonomous landing in nighttime outdoor environments with visibility constraints. The system is able to enhance the quality of the onboard-captured images effectively while performing reliable landing marker detection and validation through a hierarchical-based decision tree method and extracting the key information. Field experiments show that the vision system is accurate and robust to low illumination, motion blur, distortions, and marker occlusion. Moreover, the vision system has satisfactory timing performance and has been implemented in real-time on the onboard processing unit. Its processing speed can be further improved by utilizing the information of adjacent video frames.

At this stage, we have only shown the open-loop test results of the vision system for landing marker detection and validation, as space is limited in this paper. In the meantime, however, we are coupling the marker detection results with relative pose estimation, sensor fusion, and control system design to finalize hardware-in-the-loop field experiments. Soon, we would like to present the results of UAV fully autonomous nighttime landing and extend the existing system to land on a moving target. As the study goes on, a landing marker nighttime dataset will be released for other researchers to compare and evaluate their detection algorithms.

## Figures and Tables

**Table 1:** Statistics of the collected videos in field tests.

Scene	Luminance	Duration	Total Images	Marker Images	Max Altitude
Basketball field (L${}_{1}$)	4.0 lx	156.874 s	3116	2380	12.11 m
Warehouse (L${}_{2}$)	4.3 lx	171.416 s	3396	2437	13.51 m
Fire escape (L${}_{3}$)	4.8 lx	185.874 s	3667	2936	9.86 m
Garden (L${}_{4}$)	7.6 lx	125.657 s	2486	1888	11.08 m

**Table 2:** Number of vision system outputs at different processing stages. The terms “MK” and “BG” denote marker and background categories, respectively.

Stage	MK@L${}_{1}$	BG@L${}_{1}$	MK@L${}_{2}$	BG@L${}_{2}$	MK${}^{*}$@L${}_{3}$	BG@L${}_{3}$	MK@L${}_{4}$	MK@L${}_{4}$
ccomp	2339	5186	2396	20,149	3008	7048	1862	13,854
dt1-dt3	2291	3116	2350	3716	2964	5324	1853	4263
dt4_cnn	2252	62	2305	23	2955	92	1852	79
ext_kpt	2241	48	2218	2	2932	75	1836	65

**Table 3:** Evaluation of the proposed system using videos captured at different scenes.

Scene	TP	FP	FN	P	R	F${}_{1}$
L${}_{1}$	2241	62	139	0.973	0.942	0.957
L${}_{2}$	2218	23	219	0.990	0.910	0.948
L${}_{3}$	2852 ^∗^	92	85	0.969	0.971	0.970
L${}_{4}$	1836	79	52	0.959	0.972	0.965

**Table 4:** Evaluation of the proposed system without low-illumination image enhancement.

Scene	MK@ccomp	BG@ccomp	MK@dt4_cnn	TP	FP	FN	P	R	F${}_{1}$
L${}_{1}$	2	1589	2	2	0	2378	1.00	0.0008	0.0017
L${}_{2}$	957	1002	924	753	52	1684	0.935	0.309	0.464
L${}_{3}$	2182	2912	2095	1632	141	1305	0.920	0.556	0.693
L${}_{4}$	1372	1836	1289	886	74	1002	0.923	0.470	0.623

**Table 5:** Comparison of the CNN-based object detection frameworks.

Method	TP	FP	FN	P	R	F${}_{1}$
YOLOv3 [50]	7658	72	1484	0.991	0.838	0.91
YOLOv3-Tiny [50]	7288	34	1854	**0.995**	0.798	0.886
MobileNetv2-SSD [51]	8612	978	530	0.898	0.942	0.919
Ours	9147	256	495	0.973	**0.949**	**0.961**

**Table 6:** Evaluation of the CNN-based object detection frameworks using the enhanced image datasets.

Method	TP	FP	FN	P	R	F${}_{1}$
YOLOv3	8717	53	425	0.994	0.954	0.974
YOLOv3-Tiny	8504	26	638	**0.997**	0.930	0.962
MobileNetv2-SSD	8993	178	149	0.981	**0.984**	**0.982**

**Table 7:** Processing time evaluation on the desktop PC and TX2 unit.

Stage	HR@PC	MR@PC	LR@PC	HR@TX2	MR@TX2	LR@TX2
Image Enhancement	39.3 ms	25.3 ms	15.4 ms	94.0 ms	59.6 ms	38.2 ms
Adaptive Thresholding	14.4 ms	10.1 ms	5.6 ms	98.6 ms	76.3 ms	34.1 ms
Connected Component	2.9 ms	2.1 ms	1.3 ms	6.4 ms	4.2 ms	2.9 ms
Decision Tree Nodes 1–3	1.3 ms	1.26 ms	1.15 ms	1.81 ms	1.53 ms	1.42 ms
Decision Tree Node 4 CNN	1.4 ms	1.4 ms	1.4 ms	2.6 ms	2.6 ms	2.6 ms
Keypoint Extraction	0.02 ms	0.018 ms	0.013 ms	0.035 ms	0.033 ms	0.029 ms
Total	59.32 ms	40.18 ms	24.86 ms	203.45 ms	144.26 ms	79.25 ms
Detection Rate	16.86 Hz	24.89 Hz	40.23 Hz	4.92 Hz	6.93 Hz	12.62 Hz

## Data Availability

Not applicable.

## References

[1] Michael N., Shen S., Mohta K., Mulgaonkar Y., Kumar V., Nagatani K., Okada Y., Kiribayashi S., Otake K., Yoshida K. (2012). Collaborative mapping of an earthquake-damaged building via ground and aerial robots. J. Field Robot..

[2] Christie G., Shoemaker A., Kochersberger K., Tokekar P., McLean L., Leonessa A. (2017). Radiation search operations using scene understanding with autonomous UAV and UGV. J. Field Robot..

[3] Kerle N., Nex F., Gerke M., Duarte D., Vetrivel A. (2020). UAV-based structural damage mapping: A review. Int. J. Geo-Inf..

[4] Guo Y., Guo J., Liu C., Xiong H., Chai L., He D. (2020). Precision landing test and simulation of the agricultural UAV on apron. Sensors.

[5] Zhang G., Hsu L.T. (2018). Intelligent GNSS/INS integrated navigation system for a commercial UAV flight control system. Aerosp. Sci. Technol..

[6] Patruno C., Nitti M., Petitti A., Stella E., D’Orazio T. (2019). A bision-based approach for unmanned aerial vehicle landing. J. Intell. Robot. Syst..

[7] Yang S., Scherer S.A., Zell A. (2013). An onboard monocular vision system for autonomous takeoff, hovering and landing of a micro aerial vehicle. J. Intell. Robot. Syst..

[8] Arrar O., Aouf N., Vitanov I. (2017). Vision based autonomous landing of multirotor UAV on moving platform. J. Intell. Robot. Syst..

[9] Baca T., Petr S., Spurny V., Daniel H., Robert P., Martin S., Justin T., Giuseppe L., Vijay K. (2019). Autonomous landing on a moving vehicle with an unmanned aerial vehicle. J. Field Robot..

[10] Jin R., Owais H.M., Lin D., Song T., Yuan Y. (2019). Ellipse proposal and convolutional neural network discriminant for autonomous landing marker detection. J. Field Robot..

[11] Global Drone Regulations Database. https://droneregulations.info/.

[12] Chen X., Phang S.K., Chen B.M. System integration of a vision-guided UAV for autonomous tracking on moving platform in low illumination condition. Proceedings of the ION 2017 Pacific PNT Meeting.

[13] Meng Y., Wang W., Han H., Ban J. (2019). A visual/inertial integrated landing guidance method for UAV landing on the ship. Aerosp. Sci. Technol..

[14] Lin S., Garratt M., Lambert A. (2017). Monocular vision-based real-time target recognition and tracking for autonomously landing an UAV in a cluttered shipboard environment. Auton. Robot..

[15] Saripalli S., Montgomery J.F., Sukhatme G.S. (2003). Visually guided landing of an unmanned aerial vehicle. IEEE Trans. Robot. Autom..

[16] Lee D., Ryan T., Kim H.J. Autonomous landing of a VTOL UAV on a moving platform using image-based visual servoing. Proceedings of the 2012 IEEE International Conference on Robotics and Automation (ICRA).

[17] Serra P., Cunha R., Hamel T., Cabecinhas D., Silvestre C. (2016). Landing of a quadrotor on a moving target using dynamic image-based visual servo control. IEEE Trans. Robot..

[18] Wu Y., Niu X., Du J., Chang L., Tang H., Zhang H. (2019). Artificial marker and MEMS IMU-based pose estimation method to meet multirotor UAV landing requirements. Sensors.

[19] Masselli A., Zell A. A novel marker based tracking method for position and attitude control of MAVs. Proceedings of the International Micro Air Vehicle Conference and Flight Competition.

[20] Richardson T.S., Jones C.G., Likhoded A., Sparks E., Jordan A., Cowling I., Willcox S. (2013). Automated vision-based recovery of a rotary wing unmanned aerial vehicle onto a moving platform. J. Field Robot..

[21] Li Z., Meng C., Zhou F., Ding X., Wang X., Zhang H., Guo P., Meng X. (2019). Fast vision-based autonomous detection of moving cooperative target for unmanned aerial vehicle landing. J. Field Robot..

[22] Stepan P., Krajnik T., Petrlik M., Saska M. (2019). Vision techniques for on-board detection, following, and mapping of moving targets. J. Field Robot..

[23] Tzoumanikas D., Li W., Grimm M., Zhang K., Kovac M., Leutenegger S. (2019). Fully autonomous micro air vehicle flight and landing on a moving target using visual–inertial estimation and model-predictive control. J. Field Robot..

[24] Horla D., Giernacki W., Cieślak J., Campoy P. (2021). Altitude measurement-based optimization of the landing process of UAVs. Sensors.

[25] Nguyen P.H., Arsalan M., Koo J.H., Naqvi R.A., Truong N.Q., Park K.R. (2018). LightDenseYOLO: A fast and accurate marker tracker for autonomous UAV landing by visible light camera sensor on drone. Sensors.

[26] Yu L., Luo C., Yu X., Jiang X., Yang E., Luo C., Ren P. (2018). Deep learning for vision-based micro aerial vehicle autonomous landing. Int. J. Micro Air Veh..

[27] Abu-Jbara K., Alheadary W., Sundaramorthi G., Claudel C. A robust vision-based runway detection and tracking algorithm for automatic UAV landing. Proceedings of the 2015 International Conference on Unmanned Aircraft Systems (ICUAS).

[28] Hecker P., Angermann M., Bestmann U., Dekiert A., Wolkow S. (2019). Optical aircraft positioning for monitoring of the integrated navigation system during landing approach. Gyroscopy Navig..

[29] Hiba A., Gáti A., Manecy A. (2021). Optical navigation sensor for runway relative positioning of aircraft during final approach. Sensors.

[30] Kong W., Zhou D., Zhang Y., Zhang D., Wang X., Zhao B., Yan C., Shen L., Zhang J. A ground-based optical system for autonomous landing of a fixed wing UAV. Proceedings of the 2014 IEEE/RSJ International Conference on Intelligent Robots and Systems (IROS).

[31] Kong W., Hu T., Zhang D., Shen L., Zhang J. (2017). Localization framework for real-time UAV autonomous landing: An on-ground deployed visual approach. Sensors.

[32] Yang T., Li G., Li J., Zhang Y., Zhang X., Zhang Z., Li Z. (2016). A ground-based near infrared camera array system for UAV auto-landing in GPS-denied environment. Sensors.

[33] Kim E., Choi D. (2016). A UWB positioning network enabling unmanned aircraft systems auto land. Aerosp. Sci. Technol..

[34] Tiemann J., Wietfeld C. Scalable and precise multi-UAV indoor navigation using TDOA-based UWB localization. Proceedings of the 2017 International Conference on Indoor Positioning and Indoor Navigation (IPIN).

[35] Pavlenko T., Schütz M., Vossiek M., Walter T., Montenegro S. Wireless local positioning system for controlled UAV landing in GNSS-denied environment. Proceedings of the 2019 IEEE 5th International Workshop on Metrology for AeroSpace (MetroAeroSpace).

[36] Silva J., Mendonca R., Marques F., Rodrigues P., Santana P.S., Barata J. Saliency-based cooperative landing of a multirotor aerial vehicle on an autonomous surface vehicle. Proceedings of the 2014 IEEE International Conference on Robotics and Biomimetics (ROBIO).

[37] Sanchez-Lopez J.L., Pestana J., Saripalli S., Campoy P. (2014). An approach toward visual autonomous ship board landing of a VTOL UAV. J. Intell. Robot. Syst..

[38] Wang L., Bai X. (2018). Quadrotor autonomous approaching and landing on a vessel deck. J. Intell. Robot. Syst..

[39] Xu Z.C., Hu B.B., Liu B., Wang X., Zhang H.T. Vision-based autonomous landing of unmanned aerial vehicle on a motional unmanned surface vessel. Proceedings of the 2020 39th Chinese Control Conference (CCC).

[40] Wu S., Zhang K., Li S., Yan J. (2020). Learning to track aircraft in infrared imagery. Remote Sens..

[41] Hrúz M., Bugaj M., Novák A., Kandera B., Badánik B. (2021). The use of UAV with infrared camera and RFID for airframe condition monitoring. Appl. Sci..

[42] Xu G., Qi X., Zeng Q., Tian Y., Guo R., Wang B. (2013). Use of land’s cooperative object to estimate UAV’s pose for autonomous landing. Chin. J. Aeronaut..

[43] Kalinov I., Safronov E., Agishev R., Kurenkov M., Tsetserukou D. High-precision UAV localization system for landing on a mobile collaborative robot based on an IR marker pattern recognition. Proceedings of the 2019 IEEE 89th Vehicular Technology Conference (VTC2019-Spring).

[44] Gui Y., Guo P., Zhang H., Lei Z., Zhou X., Du J., Yu Q. (2013). Airborne vision-based navigation method for UAV accuracy landing using infrared lamps. J. Intell. Robot. Syst..

[45] Dong X., Wang G., Pang Y., Li W., Wen J., Meng W., Lu Y. Fast efficient algorithm for enhancement of low lighting video. Proceedings of the 2011 IEEE International Conference on Multimedia and Expo.

[46] He K., Sun J., Tang X. (2011). Single image haze removal using dark channel prior. IEEE Trans. Pattern Anal. Mach. Intell..

[47] He K., Zhang X., Ren S., Sun J. Deep residual learning for image recognition. Proceedings of the 2016 IEEE Conference on Computer Vision and Pattern Recognition (CVPR).

[48] Simonyan K., Zisserman A. Very deep convolutional networks for large-scale image recognition. Proceedings of the 3rd International Conference on Learning Representations.

[49] Iandola F.N., Han S., Moskewicz M.W., Ashraf K., Dally W.J., Keutzer K. (2016). SqueezeNet: AlexNet-level accuracy with 50x fewer parameters and <0.5MB model size. arXiv.

[50] Redmon J., Farhadi A. (2018). YOLOv3: An incremental improvement. arXiv.

[51] Sandler M., Howard A., Zhu M., Zhmoginov A., Chen L.C. (2018). MobileNetV2: Inverted residuals and linear bottlenecks. arXiv.

